# IFN-γ down-regulates the PD-1 expression and assist nivolumab in PD-1-blockade effect on CD8+ T-lymphocytes in pancreatic cancer

**DOI:** 10.1186/s12885-019-6145-8

**Published:** 2019-11-06

**Authors:** Guoping Ding, Tao Shen, Chen Yan, Mingjie Zhang, Zhengrong Wu, Liping Cao

**Affiliations:** 1 Department of General Surgery, Sir Run Run Shaw Hospital, School of Medicine, Zhejiang University, Hangzhou, 310000 China; 2 Department of General Surgery, Zhejiang University Huzhou hospital (Huzhou central hospital), Huzhou, 313000 China; 3 Innovation Center for Minimally Invasive Technique and Device, Zhejiang University, Hangzhou, China

**Keywords:** Interferon-γ, Nivolumab, Programmed cell death 1 receptor, T-lymphocytes, Pancreatic cancer

## Abstract

**Background:**

Pancreatic cancer is characterized by a highly immunosuppressive tumor microenvironment and evasion of immune surveillance. Although programmed cell death 1 receptor (PD-1) blockade has achieved certain success in immunogenic cancers, the responses to the PD-1 antibody are not effective or sustained in patients with pancreatic cancer.

**Methods:**

Firstly, PD-1 expressions on peripheral CD8+ T-lymphocytes of patients with pancreatic cancer and healthy donors were measured. In in vitro study, peripheral T-lymphocytes were isolated and treated with nivolumab and/or interferon-γ, and next, PD-1-blockade effects, proliferations, cytokine secretions and cytotoxic activities were tested after different treatments. In in vivo study, mice bearing subcutaneous pancreatic cancer cell lines were treated with induced T-lymphocytes and tumor sizes were measured.

**Results:**

PD-1 protein expression is increased on peripheral CD8+ T cells in patients with pancreatic ductal adenocarcinoma compared with that in health donor. PD-1 expression on CD8+ T-lymphocytes was decreased by nivolumab in a concentration-dependent manner in vitro. IFN-γ could directly down-regulate expression of PD-1 in vitro. Furthermore, the combination therapy of nivolumab and IFN-γ resulted in greatest effect of PD-1-blockde (1.73 ± 0.78), compared with IFN-γ along (18.63 ± 0.82) and nivolumab along (13.65 ± 1.22). Moreover, the effects of nivolumab plus IFN-γ largest promoted the T-lymphocytes function of proliferations, cytokine secretions and cytotoxic activities. Most importantly, T-lymphocytes induced by nivolumab plus IFN-γ presented the best repression of tumor growth.

**Conclusions:**

IFN-γ plus a PD-1-blockading agent could enhance the immunologic function and might play a crucial role in effective adoptive transfer treatments of pancreatic cancer.

## Background

Pancreatic cancer is one of the most lethal cancers, with a 5-year survival rate of 8% [1]. The incidence increased from 2000 to 2011, and an estimated 90,100 new cases and 79,000 deaths occurred in China in 2015 [2]. Because of its insidious early symptoms, rapid progression, and lack of efficient methods for early detection, more than 50% of patients are diagnosed at an advanced stage [3]. Complete surgical resection remains the first-line treatment of this malignancy; however, the radical resection rate is no more than 20% [4]. The insensitivity to chemotherapeutic drugs and radiotherapy greatly limits treatment options [5]. Therefore, discovering novel regimens for improving the curative effect of treatments for pancreatic cancer is imperative.

Pancreatic cancer is characterized by a highly immunosuppressive tumor microenvironment and evasion of immune surveillance [6]. Based on these findings, immune-based strategies to treat pancreatic cancer are showing promise. Intrinsic immune responses to malignant neoplasms are often insufficient because of inhibitory immune regulators in the tumor microenvironment. Moreover, immunotherapies such as interleukin-2 (IL-2), adoptive cell transfer, and antibodies targeting cytotoxic T-lymphocyte–associated antigen 4 or programmed death 1 receptor (PD-1) seem promising for treating cancers [7]. Adoptive cell transfer using T lymphocytes activated in vitro is an effective strategy against cancer. Similarly, activation of T lymphocytes is independent of human leukocyte antigen, whereas the persistence of immunosuppressive molecules such as T-cell membrane protein-3, cytotoxic T-lymphocyte–associated antigen 4, and PD-1 can limit the antitumor effect of adoptive immunotherapy [8].

The PD-1/PD-L1 signaling pathway is widely considered to play a crucial role in regulating the inhibition of immune responses [9–11]. The therapeutic blockade of PD-1 can improve the efficacy of the T-cell antitumor effects and reverse its inhibition [12–14]. Furthermore, nivolumab, a humanized monoclonal antibody (mAb) targeting PD-1, is approved by the United States Food and Drug Administration for treating melanoma, non-small cell lung cancer, renal cell carcinoma, Hodgkin’s lymphoma, head and neck cancer, urothelial carcinoma, and hepatocellular carcinoma [15].

Although PD-1 blockade has achieved certain success as a monotherapy, the responses to the PD-1 antibody are not effective or sustained in a subset of patients with cancer [16, 17]. The problems that must be solved are identification of the mechanism of unresponsiveness to PD-1-blockade therapy and development of mechanism-based combination therapy. For example, mutations in the genes affecting the interferon (IFN) signaling pathway are associated with acquired resistance to the PD-1 blockade in melanomas [18]. IFN gamma (IFN-γ), the only member of the type II IFN family [19], is a crucial cytokine for innate and adaptive immunity and contributes to the antitumor immune response through its immunostimulatory and immunomodulatory effects [20, 21]. Furthermore, IFN-γ activates cytokine-induced killer cells, which are capable of lysing cancer cells [22], and the IFN signaling pathway plays an essential role in improving therapeutic responses to chemotherapy [23], radiation therapy [24], and anti-human epidermal growth receptor 2 therapy [25]. However, whether IFN-γ enhances the responses of T lymphocytes to anti-PD-1 therapy is unknown.

In the present study, we addressed the question of how to improve the blocking effect of an anti-PD-1 antibody on T lymphocytes. We found that IFN-γ was an important cytokine that prolonged the responses of T lymphocytes to the anti-PD-1 antibody. Moreover, IFN-γ facilitated the antitumor immunity of the PD-1 antibody by stimulating T-cell proliferation, increasing cytokine secretion, and increasing the cytotoxicity of T lymphocytes.

## Methods

### Peripheral T lymphocytes samples

We collected 88 peripheral T lymphocytes samples, of which 48 were obtained from pancreatic ductal adenocarcinoma (PDAC) patients (23 female and 25 male patients with a median age of 62 years; age range, 41–76 years), and 40 were obtained from healthy donors (20 female and 20 male patients with a median age of 60 years; age range, 40–79 years) at the Department of General Surgery, Sir Run Run Shaw Hospital between December 2016 and May 2017. The patients with PDAC enrolled in this study only if newly diagnosed, confirmed by pathological diagnosis and underwent radical surgery. None of them had acute or chronic infections, inflammatory processes, a history of autoimmune disease, or received radiotherapy, chemotherapy, or immunotherapy before surgery. Four of the 48 PDAC patients donated 50 ml peripheral blood for subsequent T-lymphocytes culture and biological experiments. All healthy donors and patients or their guardians provided written informed consent for scientific research statement. All experiments were approved by the Research Ethics Committee of Sir Run Run Shaw Hospital, School of Medicine, Zhejiang University. All of the research protocols were carried out in accordance with approved guidelines of the Sir Run Run Shaw Hospital, School of Medicine, Zhejiang University.

### Flow cytometry

For peripheral T-lymphocytes: EDTA-anticoagulated peripheral blood was stained with with APC-conjugated mouse anti-human CD3 antibody (BD Pharmingen), FITC-conjugated anti-human CD8 antibody (BD Pharmingen), PE-conjugated mouse anti-human CD279 antibody (BD Pharmingen) or isotype control antibodies. After incubation for 30 min at 4 °C in dark. For cultured T-lymphocytes: 2 × 10^5^ cells were stained with 5 μl fluorochrome-conjugated antibodies or isotype control antibodies mentioned above for 30 min at 4 °C in dark. Next, the cells were washed twice with cold PBS. Washed cells were assayed on an BD LSRFortessa flow cytometer (BD Biosciences). Data were analyzed using FlowJo 10.0.7 software.

### Cell culture

Peripheral blood mononuclear cells (PBMCs) were obtained from the venous blood by using a lymphocyte-separating medium (Ficoll-Paque, MP Biomedicals, Carlsbad, USA). All the PBMCs were incubated in a humidified incubator at 37 °C in 5% CO_2_ for 2 h. Then, suspended cells were separated and collected to culture. The density of cells was adjusted to 1 × 10^6^/mL with 5 ml RPMI 1640 medium (Gibco) containing 5% heat-inactivated autoserum and rhIL-2 (500 U/ml, PeproTech) and incubated in flask, in which was pre-coated with OKT3 (100 ng/ml, Miltenyi Biotec), at 37 °C in 5% CO_2_ for 24 h. Fresh IL-2 and medium were added to each group every 2 days. Human pancreatic cancer cell line PANC-1, BxPC-3 and MIAPaCa-2 were obtained from Chinese Academy of Sciences (Shanghai, China). PANC-1 cells were cultured in DMEM medium (Gibco, USA) supplemented with 4.5 g/L glucose and 10% fetal bovine serum (Gibco, USA) at 37 °C in presence of 5% CO_2_. BxPC-3 cells were cultured in RPMI 1640 medium (Gibco, USA) supplemented with 10% fetal bovine serum (Gibco, USA) at 37 °C in presence of 5% CO_2_. MIAPaCa-2 cells were cultured in DMEM medium (Gibco, USA) supplemented with and 10% fetal bovine serum (Gibco, USA) at 37 °C in presence of 5% CO_2_.

### Proliferation, viability and count

The proliferation and viability of cultured T cells were detected using the trypan blue exclusion method and the automated cell counter system (TC20 automated cell counter, Bio-Rad) according the manuscript.

### Cytokine secretions

T-lymphocytes from each group were cultured on 6-well plates at concentration of 1 × 10^6^/well for 24 h. The cytokine concentrations of IFN-γ, TNF-α and IL-2 in the supernatants were detected and quantified using Cytometric Bead Array Human Th1/Th2 Cytokine Kit II (BD Biosciences, USA) with a flow cytometry system according to the manufacturers instruction as previous research [26].

### Cytotoxic activity

PANC-1, BxPC-3 and MIAPaCa-2 cells were used as target cells, stimulated group or control group cells were used as effector cells mixed in the proportion 5:1, 10:1 and 20:1. Target cells were cultured on 96-well plates at concentration of 1 × 10^5^/ml in 0.1 ml of RPMI 1640 medium containing 10% heat-inactivated fetal bovine serum for 24 h. Effector cells were inoculated onto the culture plate according to effector/target ratio in 0.1 ml of respective medium that were described in the cell culture. Some wells containing only effector cells or target cells were set as controls. There were three parallel wells for each cell count. Culture plates were placed in a humidified incubator at 37 °C in 5% CO2 for 48 h. Next, the in vitro cytotoxicity of the cells against the pancreatic cancer cells was determined using a Cell Couning Kit 8 (CCK-8, Dojindo). Optical density (OD) of each well was read at wave length of 450 nm after 4 h incubated, and cytotoxic activity was calculated as follows: Cytotoxic activity, % = [1 - (OD_effect and target cells_ - OD_effector cells_)/OD_target cells_] × 100.

### Establishment of subcutaneous PDAC mouse

All mice were obtained from the animal unit of Zhejiang University (Zhejiang, China). BALB/C nude mice (4–6 weeks old) were used in all experiments. All animal experiments were carried out in the animal unit of Zhejiang University (Zhejiang, China) according to procedures authorized and specifically approved by the animal ethics committee of Zhejiang University (Reference Number: ZJU20170126). Mice were sacrificed by CO_2_ inhalation or cervical dislocation at desired time points. Subcutaneous PDAC mouse models were established by subcutaneous injection of BxPC-3 cells (3 × 10^6^) into the left axilla of BALB/C nude mice. Then, subcutaneous tumors with a longitudinal diameter of 1 cm were peeled from subcutaneous mouse models after sacrificed. Four mice were used in each group for ectopic studies. Tumor tissues were washed in D-Hanks’ buffer. Necrotic tissues were removed from tumors, and tumor tissues were cut into about 1mm^3^ pieces. One tumor piece was implanted in the right axilla of recipient BALB/C nude mice under anesthesia. Three days after subcutaneous implantation, the mice were subjected to adoptive transfer therapy of T cells, and the tumor growth was recorded. Tumor volumes for each mouse were monitored with a caliper, every 4 days or 7 days, by measuring in two directions (length and width). The volume was calculated as length × (width)^2^ / 2. After the experiment, after anesthesia with 100% carbon dioxide, the mice were sacrificed by CO2 inhalation or cervical dislocation, and then the tumor was removed. Immunofluorescence staining was used in these tumors to detect the infiltration of CD8+ T cells.

### Immunofluorescence staining for xenograft mouse tissues

All the transplanted tumor samples were fixed by 4% PFA at 4 °C overnight and embedded into paraffin. Paraffin-embedded tissues were sectioned into 5 μm sections. The sections were deparaffinized and rehydrated by dimethylbenzene, gradient ethanol series and double-distilled water. After washed by PBS three times for 5 min, antigen retrieval was performed by boiling the sections in citric acid buffer (PH6.0) for 15 min. Cooled sections were washed by PBS, blocked by 5% normal goat serum for 45 min, and incubated with rabbit anti-human CD8 antibody at dilution of 1:100 overnight at 4 °C. Next day, the sections were stained by Alexa Fluor 594-conjugated goat anti-Rabbit IgG antibody (ab150084, Abcam), followed by DAPI staining. Slides were observed under Zeiss Observer A1 microscope. The mean number of CD8+ T cells in four microscopic fields of 40x objective was scored independently by two authors in a blinded manner.

### Statistical analysis

Statistical analyses were performed using SPSS 23.0 (SPSS Inc.; Chicago, IL, USA). Both parametric and nonparametric analyses were applied, in which the Mann-Whitney rank sum test (Mann-Whitney U test) was used for samples on a nonnormal distribution, whereas the student’s t test was performed for samples with a normal distribution. All data are reported as mean values ± standard error of the mean. A two-sided *P*-value< 0.05 was considered statistically significant. Graphical representations were performed GraphPad Prism 6 (San Diego, CA) software.

## Results

### Expression of PD-1 on peripheral CD8+ T lymphocytes

**Fig. 1:**
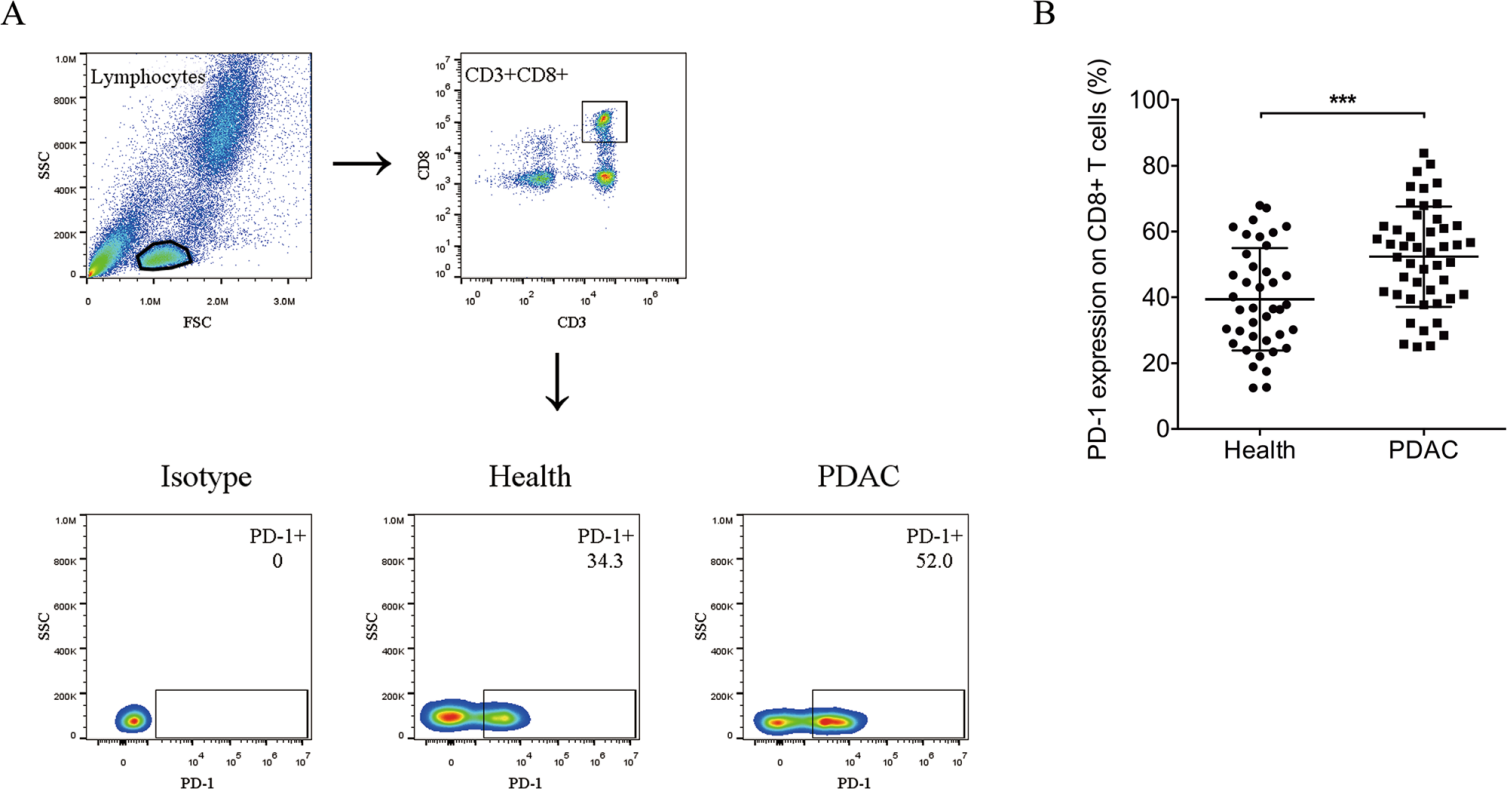
PD-1 protein expression is increased on peripheral CD8+ T cells in patients with pancreatic ductal adenocarcinoma. **a** Flow cytometry pseudo colour of lymphocytes and CD3 + CD8+ cells and representative smoothing pseudo colour of PD-1+ cells are displayed. **b** The levels of PD-1 protein expressed by peripheral CD8+ T lymphocytes in patients with pancreatic ductal adenocarcinoma (*n* = 48) and healthy donors (*n* = 40) were detected by flow cytometry. Data shown are mean ± standard deviation, two-tailed t test, *** *P* < 0.001

We used flow cytometry to compare the levels of PD-1 expression on peripheral CD8+ T lymphocytes in 48 patients newly diagnosed with pancreatic ductal adenocarcinoma (PDAC) and 40 healthy donors (Fig. 1a). PD-1 was expressed at significantly higher levels on CD8+ T lymphocytes from patients with PDAC than from healthy donors (PDAC vs. healthy donors, 52.39 ± 2.20 vs. 39.43 ± 2.45, respectively; *p* < 0.001) (Fig. 1b). These results suggest that increased expression of PD-1/PD-L1 may be associated with the evolution and progression of PDAC.

### IFN-γ down-regulates the expression of PD-1 on T lymphocytes and enhances the efficacy of anti-PD-1 therapy

**Fig. 2:**
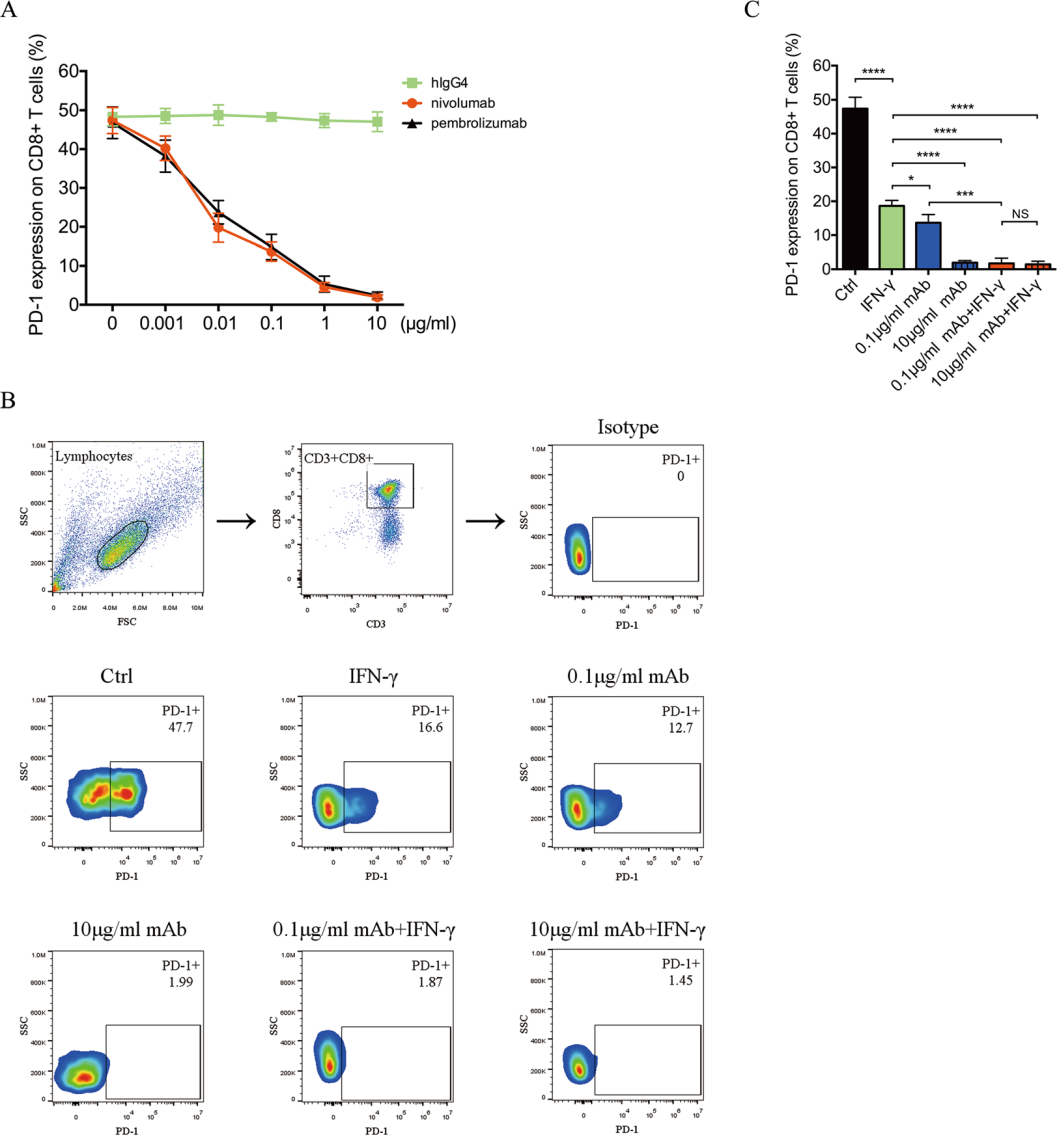
PD-1 expressions on T-lymphocytes treated with IFN-γ or/and nivolumab. **a** PD-1-blockade effect was analysed though the measurement of PD-1 expression, which is detected by flow cytometry. Compared with hIgG4 (green block), PD-1 expression was decreased along with the increase of nivolumab concentration (red point). Pembrolizumab was applied as a positive control. **b** The PD-1 expressions on CD8+ T-lymphocytes with 7 days’ treatment were measured using flow cytometry. Flow cytometry pseudo colour of lymphocytes and CD3 + CD8+ cells and representative smoothing pseudo colour of PD-1+ cells are displayed. **c** The level of PD-1 expressions was exhibited in the control group (black box), IFN-γ alone group (green box), 0.1 μg/ml nivolumab alone group (blue box), 10 μg/ml nivolumab alone group (blue stripes), combination treatment of IFN-γ and 0.1 μg/ml nivolumab group (red box), and 10 μg/ml mAb + IFN-γ group (red stripes). Data shown are mean ± standard deviation, two-tailed t test, *p < 0.05, ***p < 0.001, ****P < 0.0001, NS *p* > 0.05. Four biological replicates and three technical replicates were made in each group

To determine whether a PD-1 checkpoint-blockading agent enhances the immunological function of primary T lymphocytes from patients with PDAC, we isolated these patients’ peripheral T lymphocytes that expressed high levels of PD-1 and cultured them with different concentrations of nivolumab on the first day of induction. The T lymphocytes were treated with human immunoglobulin G4 as a negative control or with pembrolizumab as a positive control. The T lymphocytes were also treated with a CD3-activating antibody followed by the addition of recombinant human IL-2 to the culture medium. Cultured T lymphocytes were extracted on day 7, and their immune phenotypes, particularly PD-1 expression, were measured using flow cytometry. We found that PD-1 expression on CD8+ T lymphocytes decreased in a concentration-dependent manner and that the best blocking effect was achieved with 10 μg/ml of nivolumab (Fig. 2a).

**Fig. 3:**
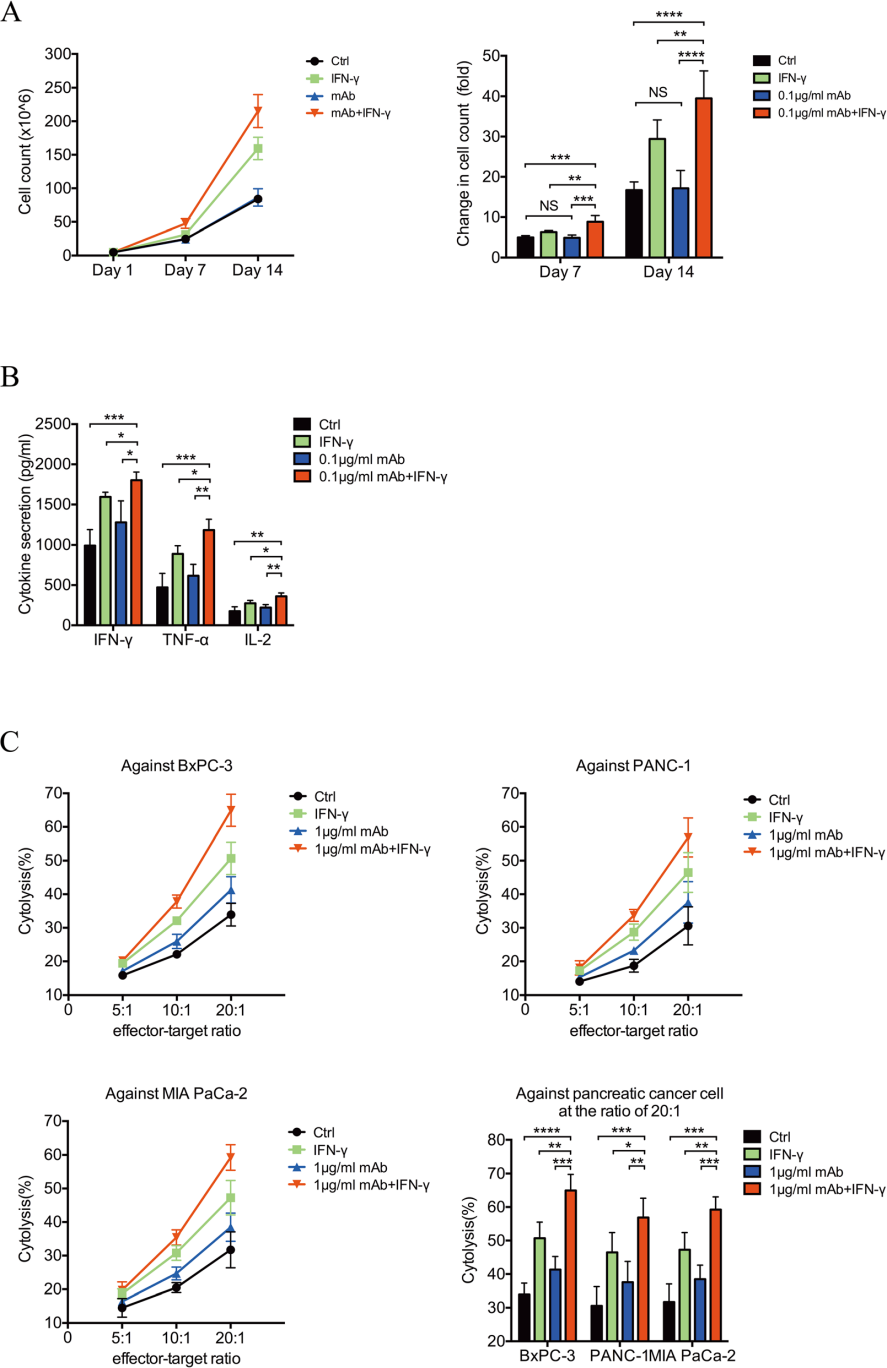
In vitro characterizations of the T-lymphocytes treated with IFN-γ or/and 0.1 μg/ml nivolumab. **a** Control T-lymphocytes (black), IFN-γ alone treated T-lymphocytes (green), 0.1 μg/ml nivolumab alone treated T-lymphocytes (blue) and combination treated T-lymphocytes (red) were stained with trypan blue, and the proliferation and viability were detected. **b** The cytokine secretions of IFN-γ, TNF-α and IL-2 from each group were measured by flow cytometry using Cytometric Bead Array Human Th1/Th2 Cytokine Kit II. **c** Pancreatic cancer cell lines such as BxPC-3, PANC-1 and MIA PaCa-2 were pre-cultured prior to the addition of IFN-γ or/and 0.1 μg/ml nivolumab treated T-lymphocytes. The cytotoxic activity of each group was determined using a Cell Couning Kit 8 and calculated as follows: Cytotoxic activity, % = [1 - (OD_effect and target cells_ - OD_effector cells_)/OD_target cells_] × 100. Data shown are mean ± standard deviation, two-tailed t test, *p < 0.05, **p < 0.01, ***p < 0.001, ****P < 0.0001, NS *p* > 0.05. Four biological replicates and three technical replicates were made in each group

As an immune adjuvant, IFN-γ enhances the immunogenicity of tumor vaccines and promotes the immune response of antigen-specific T cells. To detect the effect of IFN-γ on PD-1 expression by T lymphocytes in patients with pancreatic cancer, we added IFN-γ at a concentration of 1000 U/ml to the culture medium on day 1 during T-lymphocyte induction. T lymphocytes were isolated from the same four patients and treated with the CD3-activating antibody and recombinant human IL-2 as described above. Cultured T lymphocytes were extracted on day 7, and PD-1 expression was determined by flow cytometry (Fig. 2b). The immune phenotype of PD-1 expression of T lymphocytes was lower than that of cells not treated with IFN-γ (18.63 ± 0.82 vs. 47.38 ± 1.69, respectively; *p* < 0.0001) (Fig. 2c). However, the inhibitory effect of IFN-γ on PD-1 expression was less than that achieved with 0.1 μg/ml (18.63 ± 0.82 vs. 13.65 ± 1.22, respectively; *p* < 0.05) (Fig. 3b) or 10 μg/ml of nivolumab (18.63 ± 0.82 vs. 1.94 ± 0.31, respectively; *p* < 0.0001) (Fig. 2c).

To maximally inhibit the PD-1 checkpoint, we combined IFN-γ with 10 μg/ml of nivolumab to activate primary T lymphocytes (10-μg/ml mAb + IFN-γ group). On day 7 of culture, we used flow cytometry to detect PD-1 expression on CD8+ T lymphocytes and found that the 10-μg/ml mAb + IFN-γ group showed lower levels of PD-1 expression than the IFN-γ group (1.47 ± 0.47 vs. 18.63 ± 0.82, respectively; *p* < 0.0001) (Fig. 2c). Based on this result, we further tested the inhibitory effect of 0.1 μg/ml of nivolumab, a lower concentration, combined with IFN-γ (0.1-μg/ml mAb + IFN-γ group). Unexpectedly, we found no difference in the PD-1-blockade effect between the 0.1-μg/ml mAb + IFN-γ group and 10-μg/ml mAb + IFN-γ group (1.73 ± 0.78 vs. 1.47 ± 0.47, respectively; *p* = 0.78) (Fig. 2c). These results suggest that combination with IFN-γ allows a reduction of the dose of nivolumab and might minimize the adverse effects of nivolumab monotherapy. Moreover, the PD-1 expression was lower in the 0.1-μg/ml mAb + IFN-γ group than in the IFN-γ group (1.73 ± 0.78 vs. 18.63 ± 0.82, respectively; *p* < 0.0001) (Fig. 2c) and 0.1-μg/ml mAb group (1.73 ± 0.78 vs. 13.65 ± 1.22, respectively; *p* < 0.001) (Fig. 2c). This result further demonstrates that combining nivolumab and IFN-γ inhibits PD-1 expression greater than does nivolumab or IFN-γ as monotherapy.

### Functional analysis of induced T lymphocytes in vitro

To test the effects of IFN-γ on proliferation, cell viability, and cell density, we used the trypan blue exclusion method and an automatic counter to analyze the cells on days 1, 7, and 14. Peripheral T lymphocytes were adjusted to 1 × 10^6^/ml and 5 ml/group on day 1. During the extended culture time, the proliferation rates differed (Fig. 3a). Combined treatment with 0.1 μg/ml nivolumab and IFN-γ yielded the largest fold-increase in the number of viable cells compared with those of the control (Ctrl) group, IFN-γ group, and 0.1-μg/ml mAb group. However, there was no significant difference between the 0.1-μg/ml mAb group and Ctrl group (day 7: 4.85 ± 0.24 vs. 4.92 ± 0.10 × 10^6^, respectively, *p* = 0.79; day 14: 17.27 ± 1.30 vs. 16.82 ± 0.64 × 10^6^, respectively; *p* = 0.77) (Fig. 3a). To measure the levels of cytokines secreted from cultured cells, induced T lymphocytes were separated from each group on day 7, transferred to six-well plates, and cultured in RPMI 1640 medium without cytokines or serum; the culture supernatants were collected at 24 h after transfer. The levels of the cytokines IFN-γ, tumor necrosis factor-α (TNF-α), and IL-2 in the supernatants of all groups were quantified. The concentrations of IFN-γ, TNF-α, and IL-2 differed significantly among the groups. The highest concentrations of TNF-α, INF-γ, and IL-2 were detected in the 0.1-μg/ml mAb + IFN-γ group (Fig. 3b), suggesting that application of the anti-PD-1 antibody combined with IFN-γ at the primary stage of T-lymphocyte induction significantly up-regulated cytokine secretion.

Next, we assessed the cytotoxic activities of induced T lymphocytes in vitro. After 7 days of culture, induced T lymphocytes were collected from each group and incubated with BxPC-3, PANC-1, or MIA PaCa-2 cell lines at an effector/target ratio of 5:1, 10:1, or 20:1 (Fig. 3c). The cytotoxic activity of each group varied as a function of the effector/target ratio, and the highest lytic activity was detected using a 20:1 ratio (Fig. 3c). Further, the 0.1-μg/ml mAb + IFN-γ group exhibited the highest cytotoxic activity against pancreatic cancer cells, compared with the Ctrl group, IFN-γ group, and 0.1-μg/ml mAb group (Fig. 3c). These results suggest that nivolumab combined with IFN-γ significantly enhances the immunological functions of the T lymphocytes of patients with PDAC.

### IFN-γ improves the therapeutic efficacy of T lymphocytes blocked by PD-1 in a murine model of pancreatic cancer

**Fig. 4:**
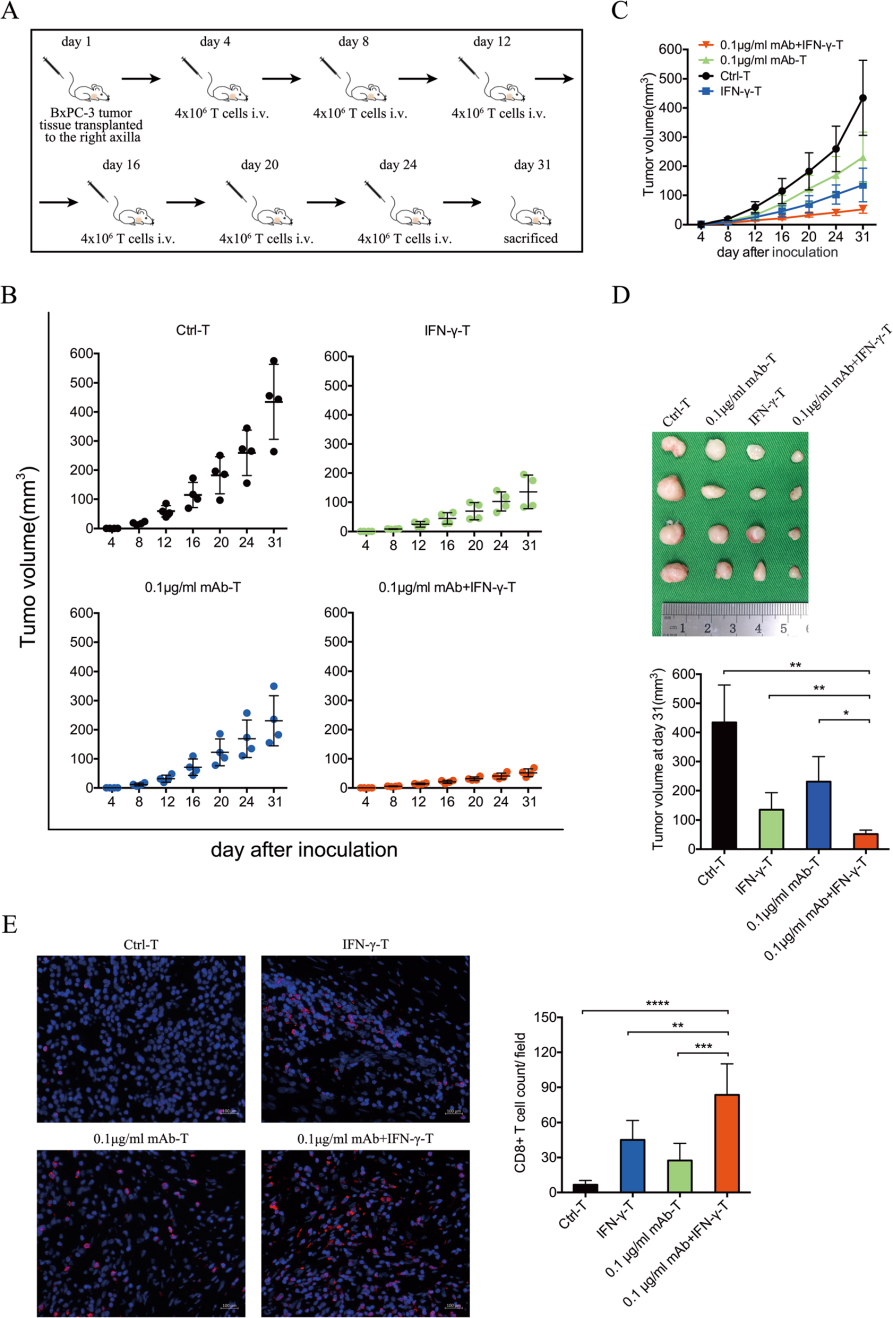
In vivo the capability of inhibiting tumor growth of the T-lymphocytes treated with IFN-γ or/and 0.1 μg/ml nivolumab. **a** Schematic diagram for the dosing regimen of T-lymphocytes treated with IFN-γ or/and 0.1 μg/ml nivolumab in subcutaneous tumor-bearing mice. **b** The capability of inhibiting tumor growth in each group throughout the treatment period. **c** Cross-comparison between mice treated with Ctrl-T, IFN-γ-T, 0.1 μg/ml nivolumab-T, or 0.1 μg/ml nivolumab+IFN-γ-T. **d** Measurement of subcutaneous tumor size at 31 days after inoculation. A significant difference was obtained between 0.1 μg/ml nivolumab+IFN-γ-T and 0.1 μg/ml nivolumab, IFN-γ-T, or Ctrl-T. Data shown are mean ± standard deviation, two-tailed t test, *p < 0.05, **p < 0.01. Four biological replicates and three technical replicates were made in each group. **e** Immunohistochemistry of CD8+ T cells in tumor sections from mice treated with Ctrl-T, IFN-γ-T, 0.1 μg/ml nivolumab-T, or 0.1 μg/ml nivolumab+IFN-γ-T (scale bar = 100 μm). Red fluorescence refers to CD8+ T cells. The number of CD8 + T cells in each microscopic field were counted for analysis. Data shown are mean ± standard deviation, two-tailed t test, **p < 0.01, ***p < 0.001, ****p < 0.0001. Four biological replicates and two immunofluorescence sections of each biological replicate were used for statistics (*n* = 8)

The tumor microenvironment plays an important role during tumor progression and treatment. The microenvironment of pancreatic cancer contributes particularly strongly to immune tolerance. To mimic this status, we tested the cultured T lymphocytes in nude mice bearing subcutaneous pancreatic cancer cell lines. The cultured T lymphocytes from each group were intravenously injected into the tail 3 days after subcutaneous implantation with BxPC-3 cells and five times at 4-day intervals thereafter (Fig. 4a). The tumor size was measured at each injection, and the last measurement was performed before sacrifice (Fig. 4b). Significant inhibition of tumor growth was observed in 0.1-μg/ml mAb-IFN-γ-treated mice compared with the IFN-γ, mAb, or Ctrl groups 31 days after the injection of BxPC-3 cells (Fig. 4c). The mean tumor sizes of the 0.1-μg/ml mAb-IFN-γ, IFN-γ, 0.1-μg/ml mAb, and Ctrl groups were 5.54 ± 0.62, 7.63 ± 0.32, 8.69 ± 0.85, and 11.06 ± 0.61 mm, respectively (Additional file 1: Figure S1). Mice treated with T lymphocytes in the 0.1-μg/ml mAb-IFN-γ group showed the highest tumor suppressive activity on day 31 after inoculation (Fig. 4d). Furthermore, immunofluorescence staining of CD8+ T cells in tumor tissues showed that the number of CD8+ T lymphocytes infiltrated in tumor tissues from the 0.1-μg/ml mAb-IFN-γ group was higher than that from the Ctrl, IFN-γ, or mAb groups (Fig. 4e). These results suggest that adoptive transfer treatment of T lymphocytes, which were stimulated by nivolumab and IFN-γ, could break through the inhibitory immune microenvironment and may therefore provide a novel approach to suppression of pancreatic cancer.

## Discussion

Pancreatic cancer remains difficult to treat, and surgical resection is the only potential therapy. However, the radical resection rate is low [4]. Local disease recurrence develops even in patients who undergo radical resection, and the relative prognosis is poor [27]. Adoptive T-lymphocyte immunotherapy has emerged as a promising approach for treating pancreatic cancer [28]. However, the presence of the immune checkpoint PD-1 on T lymphocytes and the presence of an immunosuppressive microenvironment can limit the full potential of adoptive T-cell immunotherapy. Approved PD-1 checkpoint-blockade antibodies have achieved remarkable success for treating patients with malignances such as melanoma, non-small cell lung cancer, and renal cell carcinoma [12, 13, 29]. However, they lack efficacy as single agents for immune-insensitive cancers such as pancreatic cancer. Therefore, a deeper exploration of the mechanism of insensitivity to PD-1 checkpoint-blockade agents and the development of novel mechanism-based treatments are critically important. Defects in the IFN signaling pathway may represent a potential mechanism underlying the insensitivity of cancers to immunotherapy [30, 31]. However, the volume of informative systematic research on the immunoadjuvant effects of IFN-γ on the PD-1 immune checkpoint in PDAC is insufficient.

In the present study, we first measured the expression levels of PD-1 on peripheral CD8+ T cells. Peripheral and tumor-infiltrating CD8 + PD-1+ T lymphocytes share certain phenotypes such as tumor antigen specificities and T-cell receptor repertoires [32]. Therefore, the measurement of peripheral CD8 + PD-1+ T lymphocytes may indicate the immune status of the tumor microenvironment. Accordingly, we measured the expression of PD-1 on peripheral CD8+ T lymphocytes and found that PD-1 expression was markedly higher in patients with PDAC than in healthy donors. This result, which is consistent with our previous research, indicates that peripheral PD-1 expression may serve as a new diagnostic marker and provides a target for PD-1 checkpoint-blockade agents for treating patients with PDAC [33].

To determine the blockade effect of the anti-PD-1 antibody on peripheral T lymphocytes from patients with pancreatic cancer, different doses of nivolumab were added to primary cultures of T lymphocytes. The T lymphocytes exhibited PD-1 blockade in a concentration-dependent manner, which is consistent with other studies of the properties of nivolumab in vitro [34]. IFN-γ is associated with enhanced efficacy of anti-PD-1 antibodies [35]. Therefore, we used IFN-γ to stimulate peripheral T lymphocytes in the presence or absence of nivolumab. Blockade of PD-1 occurred when the T lymphocytes were cultured in the presence of IFN-γ. As expected, IFN-γ and nivolumab combination therapy achieved the greatest inhibition of PD-1 expression. Moreover, we found that there was no significant difference in the PD-1-blockade effect between 0.1 and 1 μg/ml of mAb + IFN-γ, suggesting that the combination with IFN-γ allows a reduction of the dose of nivolumab and might minimize adverse effects compared with nivolumab monotherapy.

We further found that IFN-γ was required to promote proliferation, cytokine release, and cytotoxic activities. This is in marked contrast to stimulation by a single agent, which achieved the greatest increases in IFN-γ, TNF-α, and IL-2 secretion as well as the greatest increase in T-lymphocyte proliferation and the greatest enhancement of tumor cell lytic activity in vitro. Most importantly, in adoptive transfer experiments in which T lymphocytes were first treated with a combination of agents, the immune response improved and suppressed the growth of subcutaneous pancreatic cancer cells in mice. These results indicate the potential of T lymphocytes induced by nivolumab and IFN-γ at the primary stage as a source for adoptive transfer therapy.

## Conclusions

To our knowledge, this is the first study to investigate the potency of IFN-γ in promoting an antibody-mediated PD-1-blockade of T-lymphocytes from patients with PDAC. We hypothesize that patients with PDAC may harbor mutations in the genes affecting the IFN signaling pathway, causing the failure of anti-PD-1 monotherapy, and that IFN-γ rescues this deficiency. Moreover, these results prove that the compatibility of the immunoadjuvant IFN-γ and nivolumab can enhance antitumor immunity. We hypothesize further that pretreatment with IFN-γ and a PD-1-blockading agent may play a crucial role in effective adoptive transfer treatments of pancreatic cancer, although this disease is characterized by its low immunogenicity. Hence, these results provide better therapeutic strategies for targeting PD-1-blockade in the design of combining PD-1-blockading antibody with IFN-γ, and may help guide adoptive transfer treatments in pancreatic cancer.

## Supplementary information

**Additional file 1: Figure S1.** The average tumor sizes of 0.1 μg/ml mAb-IFN-γ, IFN-γ, 0.1 μg/ml mAb and Ctrl groups on 31 days after the injection of BxPC-3 cells.

## Data Availability

All data generated or analyzed during this study are included in this published article.

## Abbreviations

CTLA4: Cytotoxic T-lymphocyte–associated antigen 4
HLA: Human leukocyte antigen
IFN-γ: Interferon gamma
IL-2: Interleukin-2
mAb: Monoclonal antibody
NSCLC: Non-small cell lung cancer
PBMCs: Peripheral blood mononuclear cells
PD-1: Programmed death 1 receptor
PDAC: Pancreatic ductal adenocarcinoma
