# Supplementary figures and images for: IFN-γ down-regulates the PD-1 expression and assist nivolumab in PD-1-blockade effect on CD8+ T-lymphocytes in pancreatic cancer
Source: BMC Cancer. 2019 Nov 6;19:1053. doi: 10.1186/s12885-019-6145-8 (PMC6836337; doi:10.1186/s12885-019-6145-8)

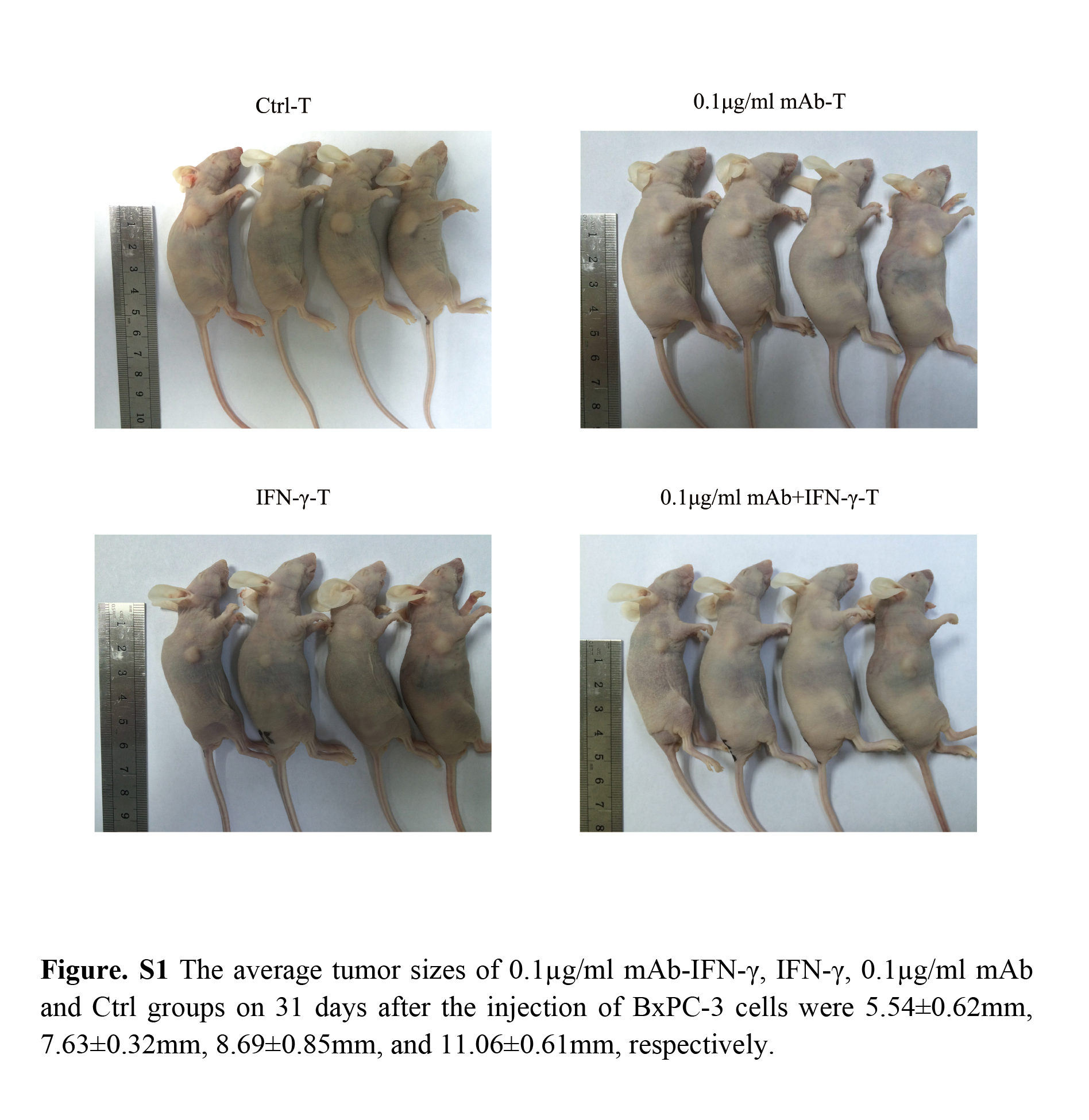

Supplement: Supplementary file 1 — Additional file 1: Figure S1. The average tumor sizes of 0.1 μg/ml mAb-IFN-γ, IFN-γ, 0.1 μg/ml mAb and Ctrl groups on 31 days after the injection of BxPC-3 cells. [file 12885_2019_6145_MOESM1_ESM.tif]
